# Unraveling the physiological roles of retinoic acid receptor-related orphan receptor α

**DOI:** 10.1038/s12276-021-00679-8

**Published:** 2021-09-29

**Authors:** Ji Min Lee, Hyunkyung Kim, Sung Hee Baek

**Affiliations:** 1 Department of Molecular Bioscience, College of Biomedical Sciences, Kangwon National University, Chuncheon, 24341 Republic of Korea; 2 Department of Biochemistry and Molecular Biology, Korea University College of Medicine, Seoul, 02841 Republic of Korea; 3 BK21 Graduate Program, Department of Biomedical Sciences, Korea University College of Medicine, Seoul, 02841 Republic of Korea; 4 Creative Research Initiatives Center for Epigenetic Code and Diseases, Department of Biological Sciences, Seoul National University, Seoul, 08826 Republic of Korea

**Keywords:** Transcriptional regulatory elements, Cell signalling

## Abstract

Retinoic acid receptor-related orphan receptor-α (RORα) is a member of the orphan nuclear receptor family and functions as a transcriptional activator in response to circadian changes. Circadian rhythms are complex cellular mechanisms regulating diverse metabolic, inflammatory, and tumorigenic gene expression pathways that govern cyclic cellular physiology. Disruption of circadian regulators, including RORα, plays a critical role in tumorigenesis and facilitates the development of inflammatory hallmarks. Although RORα contributes to overall fitness among anticancer, anti-inflammatory, lipid homeostasis, and circadian clock mechanisms, the molecular mechanisms underlying the mode of transcriptional regulation by RORα remain unclear. Nonetheless, RORα has important implications for pharmacological prevention of cancer, inflammation, and metabolic diseases, and understanding context-dependent RORα regulation will provide an innovative approach for unraveling the functional link between cancer metabolism and rhythm changes.

## Receptor proteins: adopting an orphan to understand and treat disease

The protein known as retinoic acid receptor-related orphan receptor α (RORα) activates specific genes in response to the daily cellular changes associated with circadian rhythms, and disruption of its activity is associated with cancer, inflammation and metabolic diseases. Proteins like RORα are called orphan receptors because the molecules that bind to them to initiate their activities are unknown or unconfirmed. Ji Min Lee at Kangwon National University, Chuncheon, South Korea and colleagues review current understanding of the activities of RORα, including the molecular mechanisms underlying its likely involvement in various diseases. They highlight the possible significance of manipulating RORα activity as a route towards new treatments for several diseases including cancer. Pharmaceutical companies are exploring the therapeutic possibilities of molecules that can bind to and activate RORα, especially natural products.

## Introduction

Retinoic acid receptor-related orphan receptor-α (RORα) belongs to the orphan nuclear receptor (NR) superfamily. The *RORα* gene, which is located on human chromosome 15q22.2, generates by alternative splicing four human isoforms comprising a conserved DNA-binding domain (DBD), ligand-binding domain (LBD), hinge domain, and distinct N-terminal domain. RORα is expressed in the liver, skin, lung, adipose tissue, brain, and muscle. All four isoforms, RORα1–4, are expressed in humans, whereas only RORα1 and RORα4 are expressed in mice. Expression of these isoforms varies and the isoforms regulate specific functions in several physiological processes^1,2^. The RORα recognizes a specific DNA sequence, the ROR response element (RORE), which comprises the AGGTCA consensus motif followed by an A/T-rich region, to regulate target gene transcription via interaction with coactivators and corepressors^3–5^. For most NRs, ligand binding is a critical step that triggers a conformational change in the receptor and the possibility of being replaced by a coactivator. Although cognate endogenous ligands of RORα have not been clearly identified, cholesterol and cholesterol intermediates have been suggested. Crystal structure analysis has shown that these ligands reversibly bind to RORα, but whether cholesterol and cholesterol intermediates are relevant physiological ligands remains unclear. In addition to natural ligands, SR3335 and SR1078 constitute developed inverse agonists of RORα. Selective synthetic SR3335 inhibits expression of RORα target genes, such as the gluconeogenic enzymes *G6pc* and *Pepck*^6^, and effects of SR1078 on p53 regulation, adipose tissue inflammation, renal ischemia, and allergic asthma have been studied^7–10^.

The naturally occurring mutant mouse strain “staggerer” (also known as *Rorα*^*sg/sg*^)^11^ was discovered by positional cloning^12^; it lacks up to 90% of the Purkinje cells (PCs) found in wild-type (WT) mice and has a short lifespan. The defective phenotype of staggerer mice is due to deletion of the LBD region of the RORα gene, which causes a shift in the reading frame and prevents adequate translation. These mice exhibit ataxia, motor deficiencies, irregular circadian rhythm, hyperinflammation, osteopenia, atherosclerosis, and muscle atrophy. Moreover, the similar phenotypes of RORα null-mutant (RORα^−/−^)^13^ and *Rorα*^*sg/sg*^ indicate that RORα is an essential regulator of PC differentiation and cerebellar function. Furthermore, RORα is involved in various physiological processes, such as the circadian clock, inflammation, tumorigenesis, and metabolic diseases, and it is a tumor suppressor in many types of cancer. Its expression is downregulated in cancers of the skin, colon, prostate, liver, and breast, as well as in melanoma^14–19^. Decreased RORα expression correlates with cancer aggressiveness, short overall survival, and poor prognosis. In addition, RORα modulates cyclic expression of BMAL1, a key transcription factor (TF) that regulates circadian rhythms. RORα contributes to the inflammatory response, M1/M2 polarization, and the nuclear factor-κB (NF-κB) pathway^20–22^ and regulates lipid and glucose metabolic gene expression, implying its involvement in energy homeostasis.

The regulatory mechanisms of RORα in many physiological processes are bidirectional. Its *cis*-acting regulation is based on direct recruitment of RORα to the RORE of target gene promoters. However, RORα often affects transcriptional programs in transaction mode through the antagonistic mechanisms of other TFs along with altered coregulator recruitment^15,23^. This review summarizes and discusses the current understanding of RORα function and molecular mechanisms in various pathophysiological processes, including cancer, inflammation, cerebellar development, circadian rhythm, and lipid homeostasis. This review also highlights potential targets for treating various diseases.

## Anticancer mechanisms involving RORα

Oncogenes are abnormally activated during tumor progression, which might involve signal-dependent determination of target gene expression profiles^24–27^. Cancer is associated with gene mutations, abnormal gene expression due to gene duplication, unbalanced numbers of chromosomes, or chromatin remodeling. These aberrant genetic changes can increase expression of oncogenes encoding tumor-promoting proteins or decrease that of genes encoding tumor-suppressor proteins^28–30^. Gene mutations and altered gene expression are generally connected. For instance, a gene mutation that activates an oncogene functions as a negative regulator of tumor-suppressor gene expression, which leads to a decrease in tumor suppressors. Therefore, understanding gene mutations and changes in expression is important to fully comprehend cancer.

**Fig. 1:**
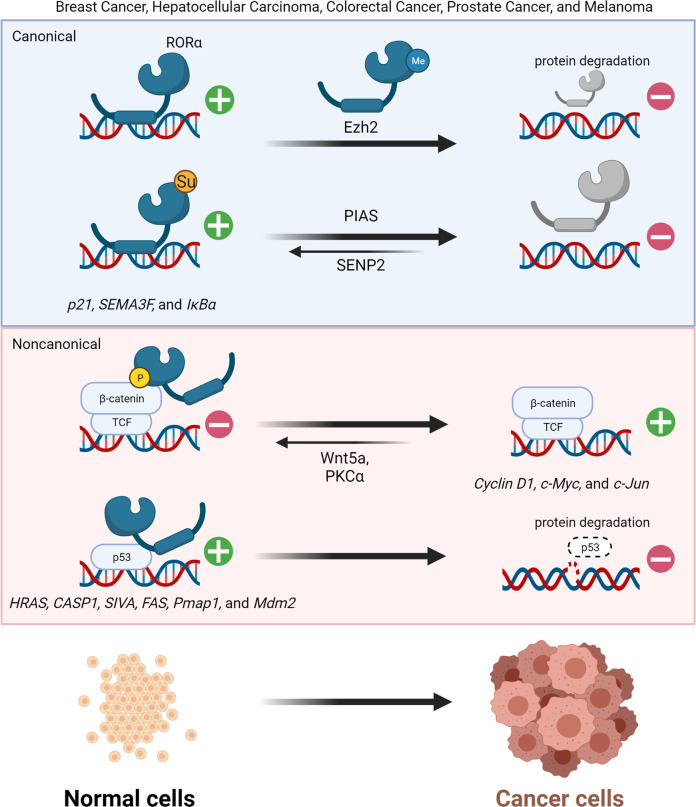
Regulatory mechanism of RORα through canonical and noncanonical pathways in cancer. Posttranslational modification by SUMOylation stimulates RORα to function as a direct TF on target gene promoters. Phosphorylation alters the direction of RORα functioning as a coregulator. Different signals can also change the roles of RORα-transregulating activities, leading to recruitment of p53-dependent target gene promoters as coactivators.

TFs interact with large complexes of coregulators in a gene-specific manner. Specific sets of TFs and coregulators are required to control subsets of genes in a given cell type. Impaired physiological pathways are important in the progression of cancer and a link between TFs and coregulators in the regulation of specific physiological pathways has been investigated^31,32^. However, under specific signaling, TFs indirectly regulate the expression of other target genes through protein–protein interactions as coregulators and this type of regulation selectively determines which genes in tumorigenesis or tumor-suppressive pathways are expressed. Increases or decreases in posttranslational modifications (PTMs), PTM crosstalk, changes in binding partners within complexes, nongenomic actions, and regulation by nuclear export signals can lead to RORα functioning as a TF that directly regulates target genes via its DBD and as a coregulator via protein–protein interactions in noncanonical pathways (Fig. 1). This type of transregulation by RORα as a coregulator allows for more sophisticated anticancer regulation than that achieved solely by increased RORα expression.

Target genes associated with several diseases, including cancer, are transcriptionally regulated by RORα. Transcriptional regulation via PTM mediated by RORα is affected by various cellular signaling pathways^33^. SUMOylation is a PTM in which a small ubiquitin-like modifier (SUMO) is added to target proteins, to change their binding affinity^34^. SUMO E3 ligase protein inhibitor of activated STAT SUMOylates RORα, whereas SUMO-specific peptidase 2 deSUMOylates it. SUMOylation upregulates RORα transcriptional activity and activates tumor-suppressive target genes^35^.

The classical ability of RORα to regulate target gene expression is key for activation of tumor-suppressive target genes (Fig. 1)^36^, although a transcription-independent function of RORα via a noncanonical pathway can potentiate tumor-suppressive roles. These functions involve direct interaction of RORα with molecules in the Wnt/β-catenin pathway, allowing RORα to transrepress Wnt pathways in colorectal cancer^22^. The Wnt signaling pathway can be classified as canonical Wnt/β-catenin or noncanonical Wnt/Ca^2+^ signaling^37,38^. As a corepressor, RORα is responsible for downregulating expression of Wnt/β-catenin target genes. In the noncanonical Wnt pathway, Wnt5a activates protein kinase Cα (PKCα) via phosphorylation and phosphorylation of RORα by activated PKCα can serve as an intersection between the noncanonical and canonical Wnt pathways. Phosphorylated RORα transrepresses β-catenin in the canonical Wnt/β-catenin pathway and downregulates expression of *cyclin D1*, *Axin*, *c-Jun*, and *c-Myc*^15^. In addition, RORα contributes to the intracellular DNA damage response, which is a checkpoint that determines cell fate, progression to a tumor or cell cycle blockade, and apoptosis during the early stage of tumorigenesis^39^. Under normal conditions, low levels of p53 are maintained by E3 ubiquitin ligases, such as Mdm2, but p53 expression is stabilized during DNA damage responses^40,41^. Moreover, expression of RORα is increased by the DNA damage signal and it is then recruited to specific genomic sites by interacting with p53 as a coregulator. The target genes of p53 comprise those with HAUSP (herpes virus-associated ubiquitin-specific protease)-dependent (*Hras*, *Casp1*, and *Siva*) and -independent (*Fas*, *Pmap1*, and *Mdm2*) promoters, with RORα functioning as a coactivator for both types^42^.

The mechanisms through which RORα acts as a canonical TF via its DBD and coregulator differ considerably. The action of RORα can be determined by the types of PTMs involved, such as SUMOylation or phosphorylation. The fact that RORα regulates expression of the master tumor-suppressor p53 and increases p53 protein stability suggests that agonists enhance the effects of RORα and create synergistic effects in two layers of RORα downstream signaling.

## Anti-inflammatory mechanisms involving RORα

Acute and chronic inflammation are major causes of cancer and understanding of their relationships is increasing^43^. The fundamental mediators between inflammation and cancer include cytokines, noncoding RNAs, and NF-κB TFs^44^, and the effects of these mediators principally cause anti- or protumorigenic inflammatory responses^45^. NF-κB is a key mediator of inflammation and tumorigenesis, and its functional link is supported by experimental evidence explaining the mutual negative regulation of NF-κB and the tumor-suppressor RORα^22,42,46,47^.

**Fig. 2:**
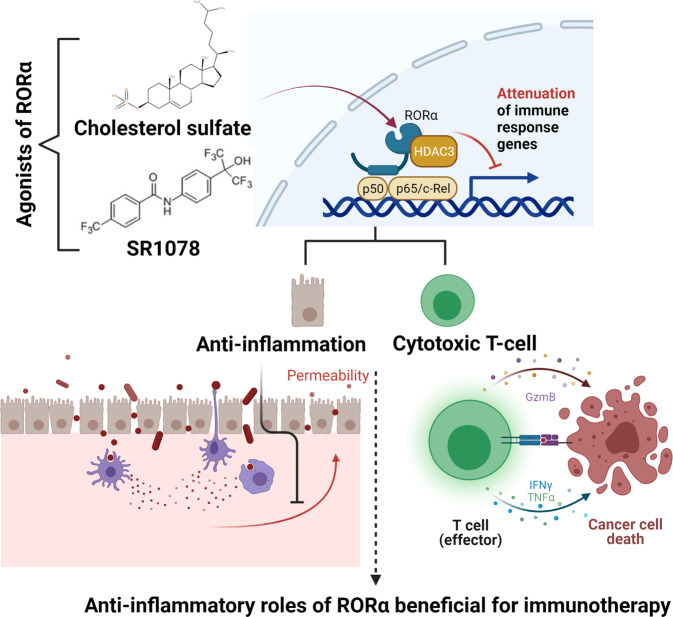
Attenuation of inflammation by RORα. Attenuated activation of NF-κB target genes by RORα contributes to survival and tumorigenesis prevention. Infectious agents lead to activation of NF-κB signaling, resulting in changes in the microenvironment to invoke immune responses and increase permeability in tissues, such as the intestine. Under ROR-mediated NF-κB target gene suppression, the functions of damaged cells are restored to reduce hyperinflammation. The anti-inflammatory roles of RORα further induce effective cytotoxic function in CD8+ T cells, resulting in cancer cell death. Activation of RORα with selective agonists, such as SR1078 and cholesterol sulfate, result in stimulatory effector responses of CD8+ T cells.

Anti-inflammatory repression through NR crosstalk (transrepression) occurs in a transacting manner without direct binding of NR to DNA response elements. Studies of RORα along with its anti-inflammatory roles in inflammatory bowel disease and ulcerative colitis have specifically advanced our understanding of the underlying molecular anti-inflammatory transrepression mechanism. The mode of action involves concomitant direct interactions of RORα with proinflammatory NF-κB and corepressor HDAC3 (Fig. 2). Recruitment of HDAC3 via RORα is responsible for the transrepressive function of NF-κB in intestinal inflammation by attenuating recruitment of histone acetyltransferase BRD4/CBP complexes and the potential application of RORα transrepression activity for therapeutics indicates new avenues for treating intestinal inflammatory diseases. The development of innovative RORα ligands would help promote anti-inflammatory effects on cancer-mediated inflammation and reduce undesirable side effects.

Metabolic changes have been observed in cancer cells and the tumor microenvironment (TME)^48^, and the mechanisms underlying these changes related to RORα have recently been identified. For example, RORα responds to metabolic changes and influences cholesterol synthesis pathways in cytotoxic T cells by inhibiting NF-κB target genes. The contributions of these activities to cytotoxic CD8+ T cells have been verified using RORα agonists^49^. Pharmacological stimulation of RORα using cholesterol sulfate and the synthetic agonist SR1078 activates downstream target genes of RORα, whereas the synthetic specific antagonist SR3335 functions as a selective inhibitor of RORα. Compared to SR1078 acting as a dual agonist of RORα and RORγ, cholesterol sulfate is a natural selective ligand of RORα. Given that directly targeting RORα via its LBD is a preferred approach for minimizing off-target effects, the selective agonist cholesterol sulfate has therapeutic anticancer effects. Cholesterol sulfate functions to regulate the effector responses of CD8+ T cells by reducing cholesterol esterification in an NF-κB suppression-dependent manner under RORα activation (Fig. 2). Immunosurveillance is an effective antitumor immune response achieved by innate and adaptive components of the immune system; however, cancer can evade immune cytotoxicity. Cytotoxic CD8+ T cells are considered the major effectors of antitumor immunity. The repressive abilities of RORα on NF-κB target genes, such as *Acat1/2* and *Abca1*, induce CD8+ T cells to secrete cytotoxic cytokines, including tumor necrosis factor-α and interferon-γ. Anti-inflammatory drugs have considerable importance for potential use in cancer chemoprevention. Indeed, anti-inflammatory drugs modulate the cytotoxic effector functions of antitumor immunity, and the attenuated inflammation and increased cytotoxic effector function promoted by activated RORα are promising for the development of a versatile therapeutic approach. Furthermore, as a safer and more effective therapy than currently available classical anti-inflammatory agents, this approach may support antitumor immune-potentiating activity because of an increased selective agonistic effect on RORα signaling in the TME.

## Control of cerebellar development and circadian rhythms by RORα

**Fig. 3:**
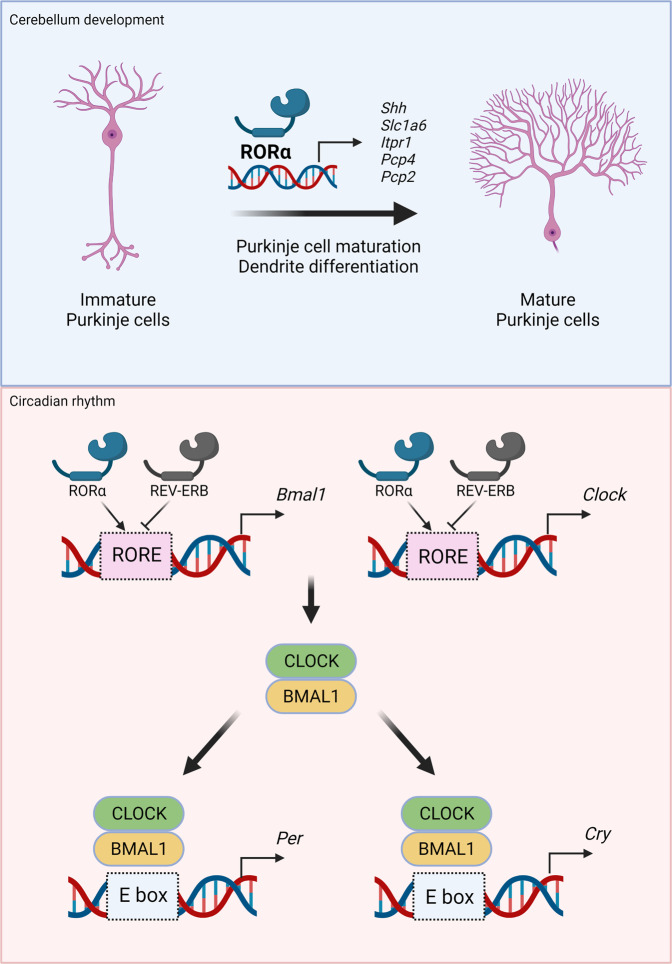
Transcriptional regulation of cerebellum development and the circadian clock via RORα and REV-ERBs. RORα controls Purkinje cell maturation through transcriptional activation of several target genes, such as *Shh*, *Slc1a6*, *Itpr1*, *Pcp4*, and *Pcp1*. Direct recruitment of RORα to target gene promoters positively regulates adequate expression for dendritic differentiation of Purkinje cells. Both RORα and REV-ERBs competitively control BMAL1 and CLOCK gene expression via RORE; RORα and REV-ERBs positively and negatively regulate BMAL1 and CLOCK, respectively. The BMAL1 and CLOCK generated cooperate at the E BOX to induce downstream target gene expression of PER and CRY, maintaining the master axis of the circadian rhythm.

Abundant RORα is expressed in cerebellar PCs, retinal ganglion cells, and thalamic nuclei. The size of the agranular cerebellum is reduced and PCs are not arranged the same way in *RORα*^*−/−*^ mice as in WT mice. The atypical cerebellar phenotypes in *RORα*^*−/−*^ and *Rorα*^*sg/sg*^ mice are identical and these defects cause abnormal cerebellar development and cerebellar ataxia because of cell-autonomous problems with PC development. Phenotypic analyses of genetically modified mice have revealed that RORα is a prerequisite for the maturation and innervation characteristics of adult PCs, suggesting that RORα is a vital determinant of survival and appropriate differentiation of PCs^50^. PCs are representative models for investigating dendrite development, as well as the function of circulating sex steroids and neurosteroids in neuronal death. Neuronal loss in *Rorα*^*+/sg*^ leads to a decline in circulating sex steroids and cerebellar neurosteroids, thus inducing PC death during normal aging^51^. Embryonic knockdown using a miRNA against RORα results in PC mislocation and primitive dendrites. These results indicate that RORα is important for PC migration and primitive dendrite regression. Therefore, appropriate expression of RORα is required for the formation and maintenance of dendrites and spines in young and adult PCs, respectively^52^. Moreover, genome-wide analysis has revealed that RORα controls the transcriptional program of cerebellar development via direct recruitment of target genes with specific coactivators, including β-catenin, p300, and tip60. After directly binding to the promoters of *Shh*, *Slc1a6*, *Itpr1*, *Pcp4*, and *Pcp2*, RORα recruits various combinations of coactivators (Fig. 3). In additon, in *RORα*^*−/−*^ mice, granular precursor proliferation and survival are rescued when recombinant proteins restore reduced *Shh* expression to normal levels^53^. Taken together, RORα maintains granular and PC functions in the cerebellum from embryonic development to adulthood.

The circadian clock is a periodic biological system that interconnects the diurnal sleep–wake cycle, metabolism, body temperature control, and immune response. Therefore, circadian rhythm dysfunction correlates with the onset of metabolic diseases, cancer, and neuropsychiatric disorders. The master axis of the circadian rhythm is generated by a specific feedback loop involving BMAL1 expression and CLOCK activation of cryptochrome (*CRY1* and *CRY2*) and period (*PER1*, *PER2*, and *PER3*) genes. BMAL1 and CLOCK TF heterodimer formation is critical for controlled expression as a negative regulatory mechanism. RORα participates in regulating the core loop by transcriptional regulation of the *BMAL1* gene with REV-ERBs^54–56^ and the dynamic interplay between RORα and REV-ERBs modulates BMAL1 expression and provides a feedback loop for timely expression. RORα works competitively with REV-ERBs by sharing the same DNA response element in the BMAL1 promoter (Fig. 3); expression of BMAL1 is silenced and increased by REV-ERBs and RORα, respectively. Oscillation by these two NRs plays a crucial role in enabling elaborate regulation of BMAL1 expression. In particular, PGC-1α is a coactivator of RORα in BMAL1 regulation; it interacts with RORα via the LXXLL motif to recruit RORE in the proximal BMAL1 promoter along with p300 and GCN5. The function of PGC-1α depends on RORE and the ability of RORα to induce BMAL1 expression is abolished in PGC-1α-null hepatocytes^57^. The CLOCK target gene depends on both RORα and REV-ERBs. Chromatin immunoprecipitation assays have revealed that RORα and REV-ERBs bind to two functional ROREs in the promoter of CLOCK and control BMAL1 expression^58^. Receptor-interacting protein 140 (RIP140) supports RORα transcriptional activity as a positive activator of the *BMAL1* gene, whereas REV-ERBs repress RIP140 expression and directly interact with RORα. As RIP140 exerts no effects in the absence of RORα, RIP140 might ROR-dependently participate in the feedback mechanism of the circadian clock and BMAL1 expression^59^.

Overall, circadian clock genes might be associated with the onset and risk of breast, prostate, and colorectal cancers and lung carcinoma^60–62^. Cancer predisposition statistically correlates with genetic variations in the circadian pathway via adaptive rank truncated product (ARTP)-based gene expression and pathways^60^. RORα ranks top among genes associated with the risk of breast and lung cancer. Moreover, RORα plays dual roles and has a functional link between circadian rhythms and cancer pathogenesis.

## Role of RORα in control of lipid homeostasis

A coordinated transcriptional network regulates expression of lipid and glucose metabolic target genes for energy homeostasis. In particular, changes in clock genes result in abnormal metabolic phenotypes in models in vivo. The roles of RORα in metabolism have been characterized in *RORα*^*−/−*^ mice. Triglyceride (TG) and total plasma cholesterol levels, as well as ApoCIII, APOA1, and APOA2 expression, are lower in *Rorα*^*sg/sg*^ mice than in WT mice. In addition, expression of the cholesterol transporters *Abca1* and *Abca8/G1* is attenuated in *Rorα*^*sg/sg*^ mice, suggesting that the phenotype of impaired high-density lipoprotein biosynthesis is caused by decreased expression of ABCA1^63^. Reduced TGs in *Rorα*^*sg/sg*^ mice are due to downregulation of transcriptional regulators responsible for fatty acid biosynthesis and lipogenesis, including SREBP1c, FAS, and ABCG1. Indeed, *Rorα*^*sg/sg*^ mice are resistant to standard high-fat diet (HFD)-induced obesity and weight gain. Although heart and liver tissue weights do not significantly differ between WT and *Rorα*^*sg/sg*^ mice, epididymal and inguinal fat depots are distinct. Moreover, PCK1 expression is reduced in the livers of *Rorα*^*sg/sg*^ mice after a 6 h fast. The gluconeogenic enzyme PCK1 is a rate-determining factor in the adaptive response of the fasting period. Hepatic gluconeogenesis and adipose tissue glyceroneogenesis are positively controlled by RORα and its regulation is mediated by PCK1^64^. Expression of thermogenic genes, such as UCP1, is increased in brown and subcutaneous inguinal adipose compartments of *Rorα*^*sg/sg*^ mice, suggesting increased thermal control and cold tolerance in these mice^65^. This process is associated with increased oxygen consumption and energy expenditure.

**Fig. 4:**
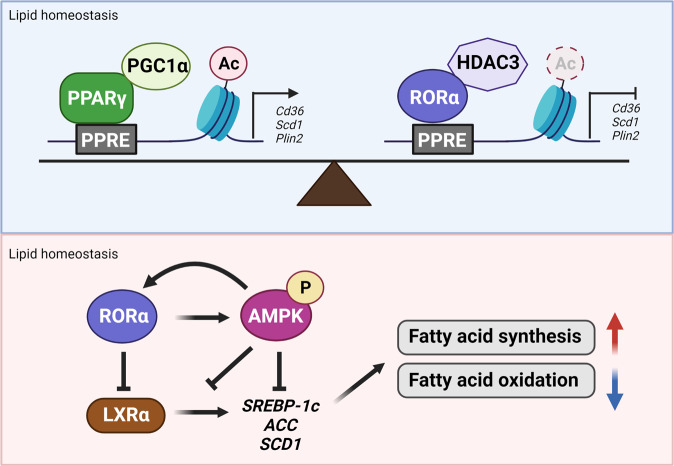
Regulation of RORα in lipid homeostasis. Transcriptional regulation of lipid homeostasis mediated by PPAR is inhibited by RORα. Transcriptional activation of PPARγ with PGC-1α and transcriptional repression of RORα with HDAC3 balance each other at the promoters of PPAR-dependent target genes. Simultaneously, AMPK activation and antagonized LXRα expression by RORα results in reduced expression of lipogenic genes, such as *SREBP-1* and *FAS*. Downregulated SREBP-1 results in decreased fatty acid oxidation and lipogenic effects.

Nevertheless, a HFD significantly increases body weight and the obesity phenotype in liver-specific *Rorα* conditional knockout mice (*RORα*^*LKO*^) compared with *RORα-*floxed mice (*RORα*^*f/f*^). White and brown fat depots and the area of adipocytes in visceral fat tissue are significantly increased in *RORα*^*LKO*^ mice, and the livers of HFD-fed *RORα*^*LKO*^ mice are enlarged and paler than those in HFD-fed *RORα*^*f/f*^ mice. Oil red O staining and hepatic TG analysis have revealed significantly increased lipid levels in HFD-fed *RORα*^*LKO*^ mice. Genes associated with lipogenesis, gluconeogenesis, and lipid sequestration are abundantly expressed in *RORα*^*LKO*^ mice fed an HFD, suggesting that RORα negatively regulates HFD-induced steatosis. Furthermore, genome-wide analysis showed that RORα is involved in PPARα-dependent target gene transcription. The target genes of PPARγ, such as *Cd36*, *Plin2*, and *Scd1*, are elevated in HFD-fed *RORα*^*LKO*^ mice compared with HFD-fed *RORα*^*f/f*^ mice. The absence of RORα leads to enhanced PPARγ recruitment to target genes and subsequent elevated expression of downstream target genes, indicating that RORα suppresses peroxisome proliferator-activated receptor-γ (PPARγ)-dependent target gene expression. In addition, RORα is directly recruited to the peroxisome proliferator-activated receptor element (PPRE) of a target gene promoter with HDAC3 for repression and competes with PPARγ (Fig. 4). Indeed, metabolic defects, such as body weight gain, hepatic steatosis, and lipid metabolism in HFD-fed *RORα*^*LKO*^ mice, are mitigated by PPARγ antagonism with GW9662. Thus, RORα is a crucial factor in maintaining diet-induced hepatic lipid homeostasis through suppression of PPARγ signaling. The phenotypes of RORα and RORγ double-knockout mice in the lipogenic response have been determined^66^, and the ROR family subtype RORγ functions in hepatic gluconeogenesis. Generating double-floxed liver-specific, double-knockout (LDKO) mice by injecting targeted AAV-TBG-Cre has revealed that although expression of circadian clock genes, such as *Bmal1*, *Cry1*, and *Npas2*, is downregulated in LDKO mice, that of *Fasn*, *Elovl6*, and *Acaca*, which are involved in hepatic lipid and fatty acid metabolism, is enhanced. Both RORα and RORγ show functional redundancy in target gene expression in the mouse liver. Moreover, RORα and RORγ control *Insig2a* expression to repress SREBP1c, which is required for cholesterol synthesis and hepatic lipid metabolism at the end of the feeding phase in the dark. Consistent with this finding, the significantly increased hepatic TG levels and intensely Oil Red O-stained liver sections in ROR LDKO mice indicate that ROR regulates the homeostasis of lipogenic metabolism. The activity of RORα toward liver X receptor α (LXRα) in hepatic steatosis is antagonistic^42^. Activation of AMPK is induced by RORα, which leads to simultaneous reductions in LXRα protein levels and inhibition of transcriptional activity (Fig. 4). As LXRα is a positive regulator of hyperglycemia and hepatic fatty acid synthesis^67^, expression of lipogenic genes, such as *Srepb-1c*, *Fas*, and *Accα*, is diminished and the antilipogenic effects of RORα have been validated in HFD-induced fatty liver. Orally administered ROR-activating compounds drive AMPK activation as well as LXRα inhibition in the liver of HFD models, decreasing TG and body weight. Moreover, RORα expression correlates with the onset of metabolic diseases such as HFD-induced nonalcoholic steatohepatitis, coronary heart disease with concomitant abdominal obesity, and metabolic syndrome^21,68–70^. Therefore, RORα may be considered a key target in the development of agents to treat various metabolic diseases.

## Conclusions

Circadian and metabolic disruptions are associated with an increased incidence of specific types of cancer and inflammatory diseases; however, the relationships between causes and mediators remain unclear^71,72^. As RORα regulates cancer, inflammation, circadian rhythm, and metabolism, it not only functions as a transcriptional effector but also as a sensitive sensor that captures and reconstructs distinct changes in the cellular environment into transcriptional responses^73^. Elucidating the signaling pathways of second messengers, hormones, and chromatin configurations, which modulate interactions between RORα and its binding partners, is important. Such knowledge is relevant to the identification and development of novel compounds that increase or decrease the binding affinity of RORα and its complexes, and direct the activity of cofactors toward disease crosstalk pathways.

**Fig. 5:**
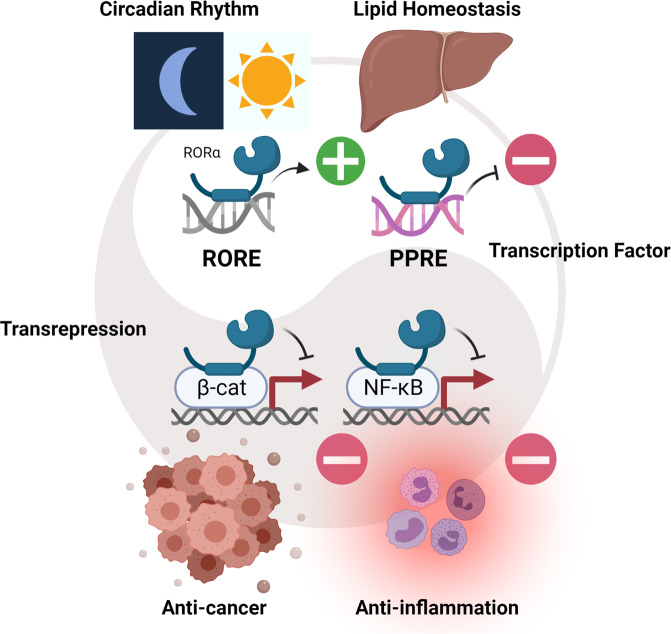
Target gene activation or repression by RORα and functions of RORα. Circadian rhythm, lipid homeostasis, anticancer, and anti-inflammatory effects are integrated by processes in which RORα plays key roles. Direct activation of RORα via RORE may result in balanced circadian clock gene expression and different responses might be repressed by the same TF through binding of different PPREs. Evidence indicates that RORα functions in transrepression through other TFs such as β-catenin and NF-κB, which are master regulators of tumorigenesis and inflammation, respectively. The roles of RORα in health and disease are likely to depend on which cellular response is switched on and the context in which target selection occurs via RORα.

The role of RORα as a TF or a coregulator is important for both fine-tuning transcriptional networks and further regulating many aspects of disease progression or suppression^74,75^. A controlled balance between direct TFs and coregulators in transcriptional programs can promote or inhibit target gene expression, and defining and harmonizing the signaling pathways that modulate gene expression between TFs and coregulators is crucial. The fact that different target genes of the same regulator RORα require distinctive combinations of TFs and coregulators is due to distinct chromatin environments and signaling cascades. The specific DNA sequence to which a TF binds can affect the selection of complex combinations and their actions. Variations in upstream signaling may influence recruitment of RORα as a coregulator. In addition, the coregulator activity of RORα may enhance other TF activities and bias expression of pathway-specific genes with unwanted side effects because of the gene- and pathway-specific actions of TF-coregulator combinations (Fig. 5).

Overall, targeting individual pathways using a direct inhibitor of RORα as a TF by suppressing the DBD or LBD only inhibits one or two pathways. Disease progression can be decelerated, but other pathways in which RORα functions as a coregulator and transregulator of target genes may be upregulated as compensation, thus generating resistance to therapy. If a defined role of RORα as a coregulator is targeted and its function prevented, combined pathways will be concurrently inhibited. Such a strategy blocks the alternate pathway of compensatory upregulation, decreasing the onset of acquired drug resistance. In this scheme, a small-molecule drug designed to block a specific action of RORα, such as a PKCα agonist acting as a coregulator, can lead to simultaneous inhibition of an array of Wnt/β-catenin downstream target genes. As PTMs designating the action of RORα as a TF or a coregulator are a result of prior specific enzymatic reactions, drugs can inhibit or activate modifying enzymes. These approaches are important for understanding the regulation of specific noncanonical pathways in which drugs do not modulate RORα via LBDs, suggesting that a mechanism-based drug administration strategy may be useful for specifically inhibiting cancer activity.

Future investigation in these areas will be critical for defining the sensory and physiological codes of the dual RORα roles as a TF and coregulator, and recognizing the potential of dual concepts as therapeutic targets. Only a few signaling pathways or PTMs that regulate the activities of specific recruitment of RORα as a direct TF or coregulator have been elucidated thus far. The specific signaling pathways and enzymes responsible for conducting the signaling cascade in terms of TFs or coregulators and the inhibitors that would be applicable for clinical applications await further investigation in the future.

Many efforts to find ligands of RORα, such as the discovery of endogenous ligands and the chemical synthesis for synthetic ligand design, have been applied to verify its importance α in various biological systems and disease models. Although T0901317, first known as an inverse agonist of RORα, was revealed to be a nonspecific ligand for several NRs, it contributed to the development of a new synthetic ligand. In addition, SR1078 showed efficacy in p53 stabilization and subsequent apoptosis, and may be utilized in cancer treatment in the future. In addition, the inverse agonist SR1001, which interferes with the interaction between receptor and coactivator, has been proven to be effective for treating autoimmune disease. Many researchers have attempted to discover a potent and selective synthetic ligand of RORα and verify its efficacy in disease models. Although RORα is a very promising target that can be applied to circadian rhythm, cancer, and metabolic and immune diseases, there are no drugs precisely targeting RORα on the market. Several pharmaceutical companies are dedicated to the discovery and development of using natural products as selective agonists of RORα. For example, GENFIT and BICOLL identified the first potent bioavailable natural product neoruscogenin as a specific agonist of RORα, and they are developing neoruscogenin as a therapeutic drug for autoimmune disorders in the preclinical stage^76^. Therefore, the development of drugs targeting RORα in the future will be a significant and valuable achievement for treating related diseases.
